# The important versus the exciting: reining contradictions in contemporary biotechnology

**DOI:** 10.1111/1751-7915.13348

**Published:** 2018-12-03

**Authors:** Víctor de Lorenzo, Jillian Couto

**Affiliations:** ^1^ Centro Nacional de Biotecnología‐CSIC Campus de Cantoblanco Madrid 28049 Spain; ^2^ School of Engineering University of Glasgow Glasgow G12 8QQ UK

The applicability agenda of modern Biotechnology needs no explanation or justification. As any technology, the ultimate value of Biotech is to deliver materials, molecules or processes of interest for the medical, agricultural and environmental market. However, if one takes a look at high‐impact journals or reads grant proposals on topics presented as Biotechnological research, one legitimately wonders when and how the many exciting results and concepts that often make headlines will get close to actual, beneficial uses to society and economy. There is indeed a *Valley of Death* in terms of funding between original scientific discoveries and applications that is typically punctuated by the scores (1–9) of the so‐called Technical Readiness Level (TLR). But – important as it is – in our opinion, the issue is not just about funding but also about overcoming the conundrum *exciting vs. important*. The first gets all the attention, receives all funding and it is frequently published in prestigious journals. No surprise that the most creative minds flock to identify and get engaged in exciting endeavours, not the least because of the career benefits involved. However, exciting seminal discoveries in Biotechnology are worth nothing if they are not followed up all the way to technological readiness. Fleming's observations on penicillin had to wait two decades until Florey and Chain figured out ways to scale‐up the production process. But who remembers the last two? In sum, it seems that finding or developing properties with a biotechnological potential is exciting. But scaling up and designing processes is perceived as boring, a sort of second‐level endeavour to be addressed also by second‐level professionals. In this Crystal Ball, we would like to take the opportunity to argue how wrong this is.

Scale‐up is in fact the most important bottleneck that contemporary Biotech has to address if it is to find its niche in a large‐scale industrial landscape. This is different from the small‐scale production of very high added value biomolecules, such as pharmaceuticals, and the GMO‐based agriculture. Let us take an example close to the core Microbial Biotech agenda: whole‐cell catalysis and biotransformations. The literature has plenty of metabolic engineering cases where microbial strain A (or even an artificial consortium) is heavily refactored genetically by scientist B to produce compound C (biopolymers, biofuels, fragrances, food additives, speciality chemicals etc.). Although the yields are typically not great, the high‐prominence work on this project done by researcher B often stops there (the generation of a new property). What happens next (if it does) generally disappears from the high‐visibility radar and B moves on to engineer another strain A that produces another interesting molecule C and so on. The scale‐up and the downstream processing (the most limiting factors for raising industrial interest) are taken for granted, handed over to engineers and generally considered devoid of much interest. This state of affairs creates a scenario in which genetic and metabolic engineering of biological systems advance at the speed of light. But process engineering seems to be stuck in century‐old principles.

The iconic and still prevailing production setup for biomolecules synthesized by microorganisms is that of a fed‐batch fermentor with sizes anywhere between millilitres to cubic metres, inoculated with a single strain and grown in a sterile culture medium. After fermentation, the biomass has to be separated, the product of interest extracted and the liquid medium disposed of as waste. While much of the progress in reactor design has focused on automation and control, in reality the principles behind such fed‐batch fermentations are not that different from ancient Egyptians producing beer thousands of year ago! Can we do better? The answer is yes, provided that we reformulate what appears to some like mere technicalities into exciting research issues. Let us just comment on a few of these questions, the first of which is the genetic design of whole‐cell catalysts.

For example, we still know very little on how microorganisms adapt to living in a bioreactor. It is important to note that this is an artificial niche and the biological agenda of the catalyst (as in any other place) is about surviving and thriving in such an unusual scenario. Thus, in addressing this relationship, the interplay between the fundamentals of ecology (the niche) and evolution (how the catalyst adapts), becomes highly relevant to this prosaic industrial process. One conveniently ignored issue is that the fermentor niche is known to be fluctuating as it is impossible to fully mix large‐scale industrial reactors to virtual homogeneity. This creates zones where glucose limitation, pH fluctuations and oxygen restriction are a common occurrence and can lead to physiological stress placed on the production organism (Glazyrina *et al*., 2010; Neubauer and Junne, 2010). Recent studies on long‐term residence of *E. coli* in a continuous reactor have revealed a suite of genes of unknown function that get turned on and off (and even deleted) under such conditions (Loffler *et al*., 2016) demonstrating the impact of fermentor ecology on evolution. Therefore, this change of biological agenda in response to localized changes in the environments that the whole catalysts go through points to the importance of gene–environment interactions and is something that should be considered during genome design. Up until now, design strategies are heavily directed to reducing metabolic burden, which is an important consideration (Borkowski *et al*., 2016; Ceroni *et al*., 2016). Also, the genomes of industrial strains have been edited to reinforce stress resistance and strengthen metabolic capacity by, for example removing ATP and NADH‐consuming functions (Lieder *et al*., 2015). However, genome‐reduced strains originally created for increasing predictability of biotechnological processes turned out to fare worse than non‐reduced counterparts in environments that recreate stresses typical of these industrial transformations (Couto *et al*., 2018). Thus, genome reduction strategies that exclusively focus on metabolic burden might have unexpectedly led to the removal of genes that enable the organism to respond to its changing environment. To remedy this, the genome designer needs to strike a balance between reducing metabolic burden and enabling the organism to persist in the process environment. It is possible that to enable this one must consider the *minimal gene network* required for robust growth instead of the *minimal number of genes* for robust growth. Critically, this *gene network* might only become obvious in growth conditions the catalyst will face during the process, as opposed to in ideal Laboratory conditions, and we must incorporate this into our routine experimental procedures.

A second issue is that of large volumes of liquid needed for the culture medium in fermentations. With the exception of marine and freshwater microorganisms, most bacteria habitually live and thrive under sub‐saturating humidity conditions. Why do we need to use so much water in our fermentations, which complicates downstream processing and generates much waste? One way to go is developing high‐density processes or even slurry‐based processes. But the real breakthrough would be having transformations occurring with no or very little added water, that is a sort of composting or solid‐phase fermentations (Arora *et al*., 2018). We have precedent here as anaerobic digestion (AD), a biological process that is now a firmly established energy‐from‐waste biotechnology is known to be most efficient with high‐solid/low water‐containing waste streams. Importantly, although this process has been in use since the 16th century, the reactor has gone through various design iterations on its journey from early small‐scale operations to biogas refinery. Unlike fermentations, AD retains the biomass as granular biofilms that mix with feedstock for a set hydraulic retention time, during which organic materials are metabolized, leaving less harmful products. The chemicals of interest could then be engineered for being secreted – thereby negating the need for breaking up the biomass for extractions. Simultaneously, reactors could be redesigned to contain a series of membrane structures or scaffolds that house or retain this biomass within the reactor. In this case, there is much inspiration to get – once again – from naturally occurring systems. Instead of one‐pot reactors, new settings could be entertained mimicking a large‐scale, metabolically super‐active multicellular organism such as a rubber tree or an organ such as an udder where simple feedstocks diffuse through the system for a set hydraulic retention time during which the bioconversion to valuable chemicals takes place. Note also that such *natural reactors* operate without sterilization and deliver products nearly ready to use. There is much room for improving this feature as well. As proposed recently one can design large‐scale production of bioplastics in non‐sterile conditions by growing engineered *Halomonas* sp. in seawater, a niche that can hardly be contaminated by non‐marine bacteria (Chen *et al*., 2018). We do not wish to annoy our dear bioprocess engineering colleagues, but we cannot avoid the feeling that the field has been far more conservative and less creative than the coetaneous advances in the genetic design of biological catalysts brought about by contemporary Systems and Synthetic Biology!

In sum, in this CB we envision that for the so‐called 4th Industrial Revolution (Schwab, 2017) to equal the impact of its predecessors in the Biotechnological realm, a radical redesign of the bioprocess requires us to go back to the fundamentals of ecology and evolution as well as creatively rethinking both the organism and its environment. And in asking these fundamental questions we place ourselves in the position of doing exciting research while at the same addressing important problems.
